# Author Correction: Cryo-EM structure of the bacterial Ton motor subcomplex ExbB–ExbD provides information on structure and stoichiometry

**DOI:** 10.1038/s42003-020-01427-w

**Published:** 2020-11-09

**Authors:** Herve Celia, Istvan Botos, Xiaodan Ni, Tara Fox, Natalia de Val, Roland Lloubes, Jiansen Jiang, Susan K. Buchanan

**Affiliations:** 1 Laboratory of Molecular Biology, National Institute of Diabetes and Digestive and Kidney Diseases, National Institutes of Health, Bethesda, MD 20892 USA; 2 Laboratory of Membrane Proteins and Structural Biology, Biochemistry and Biophysics Center, National Heart, Lung, and Blood Institute, National Institutes of Health, Bethesda, MD 20892 USA; 3 Center for Molecular Microscopy, Center for Cancer Research, National Cancer institute, National Institutes of Health, Bethesda, MD 20892 USA; 4 Cancer Research Technology Program, Frederick National Laboratory for Cancer Research, Leidos Biomedical Research Inc, Frederick, MD 21701 USA; 5 Laboratoire d’Ingénierie des Systèmes Macromoléculaires, UMR7255 CNRS/Aix-Marseille Université, Institut de Microbiologie de la Méditerranée, 13402 Marseille Cedex 20, France

**Keywords:** Cryoelectron microscopy, Bacterial structural biology

Correction to: *Communications Biology* 10.1038/s42003-019-0604-2, published online 4 October 2019.

In the original version of the published article, the caption to Figure 2e contained the following text that incorrectly referenced ExbB and an incorrect PDB code: “Ribbon representation of the solution structure of the ExbB soluble periplasmic domain (in green, pdb code 2PDU)^4^ and ExbB–ExbD”.

This has been replaced with the following corrected text: “Ribbon representation of the solution structure of the ExbD soluble periplasmic domain (in green, pdb code 2PFU)^4^ and ExbB–ExbD”. The error has been corrected in the HTML and PDF versions of the article.

