# Supplementary material for: Cryo-EM structure of the bacterial Ton motor subcomplex ExbB–ExbD provides information on structure and stoichiometry
Source: Commun Biol. 2019 Oct 4;2:358. doi: 10.1038/s42003-019-0604-2 (PMC6778125; doi:10.1038/s42003-019-0604-2)
Supplement: Supplementary file 1 — Supplementary Information [file 42003_2019_604_MOESM1_ESM.pdf]

Supplementary Figure 1

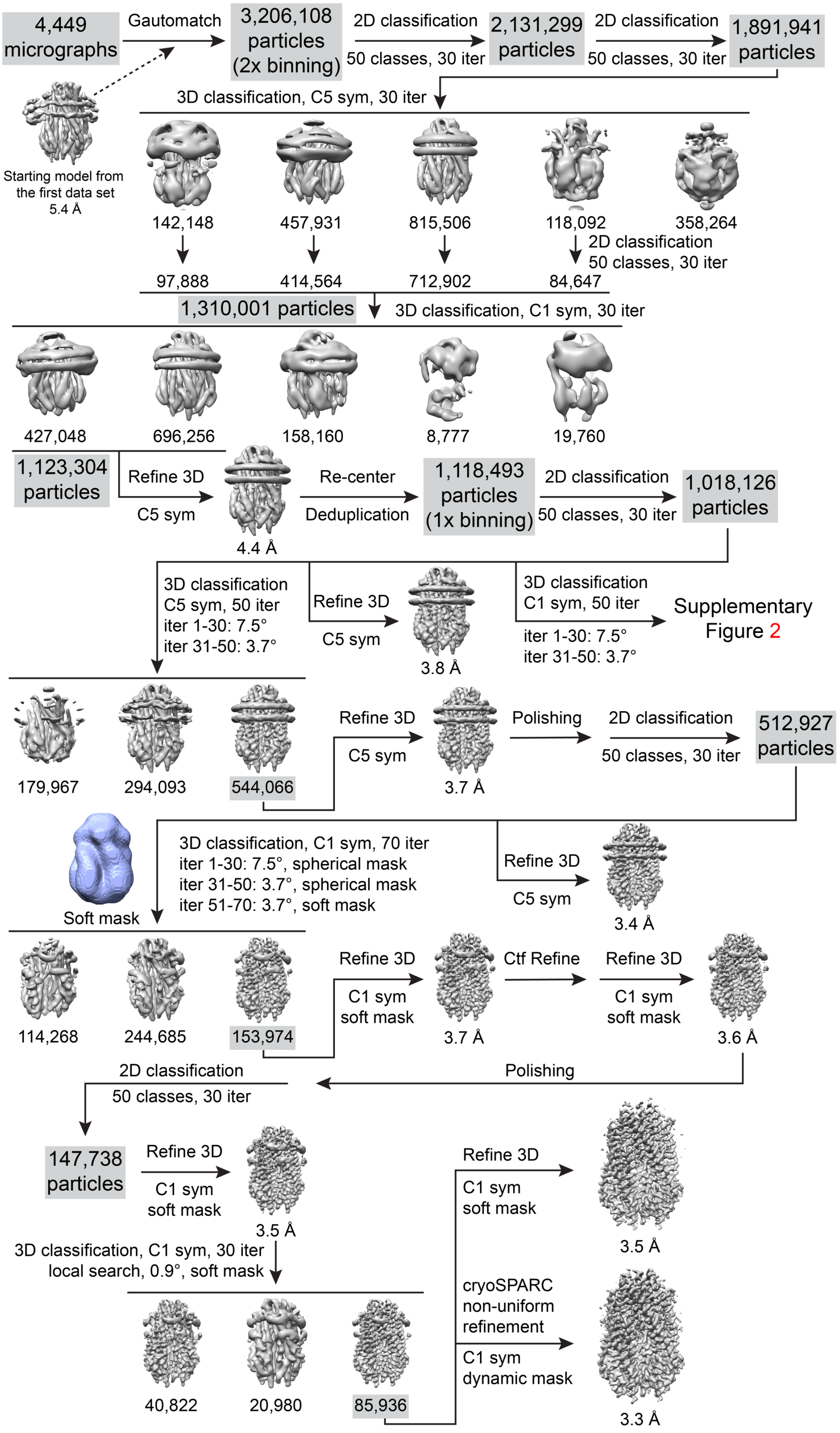

Schematic diagram of the cryoEM data processing procedures.

Supplementary Figure 2

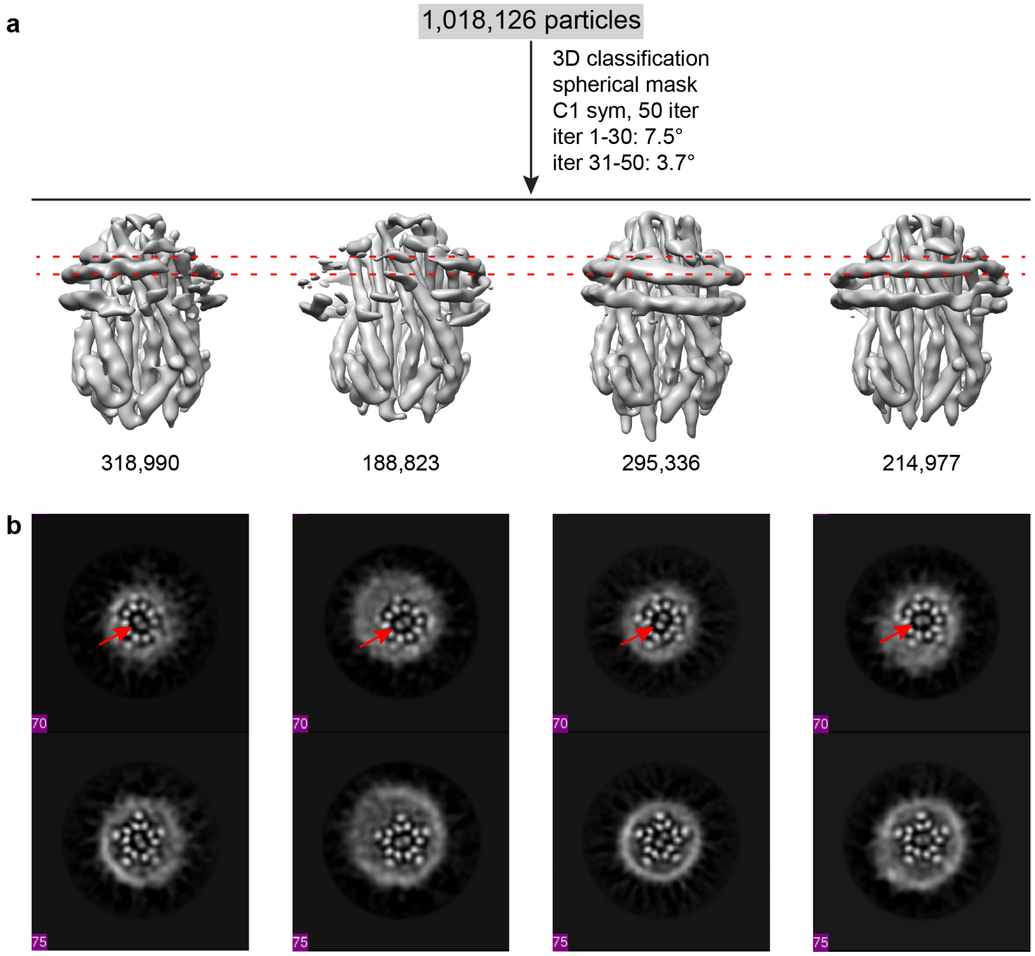

RELION 3D classification with C1 symmetry reveals densities of two helices in the pore of the ExbB pentamer. a. 3D reconstruction of four classes of particles separated by 3D classification with C1 symmetry and a spherical mask. The red dotted lines indicate the positions of the cross sections shown in (b). The number of particles (1,018,126) used for this 3D classification is about 32% of the total particles (3,206,108) picked from the micrographs. The rest of particles (68%) were removed by 2D classification and 3D classification (see Methods and Supplementary Figure 1) because the corresponding classes had poor and uninterpretable features, suggesting they were damaged, improperly assembled, or not real particles. The above selections were not based on conformational variances. Therefore, the 32% population of total particles used for this 3D classification can represent the conformational distribution in the sample used for this study. b. Two cross-sectional views (slices 70 and 75 of a volume of 192 slices) of the 3D reconstruction of each 3D class. The red arrows point to the densities corresponding to two helices in the pore. It is worth noting that the conformation of the ExbB-ExbD complex (the dots in pentagonal arrangement) remains nearly unchanged while the structure of the lipid nanodisc (circular cloudy densities) is remarkably variable.

Supplementary Figure 3

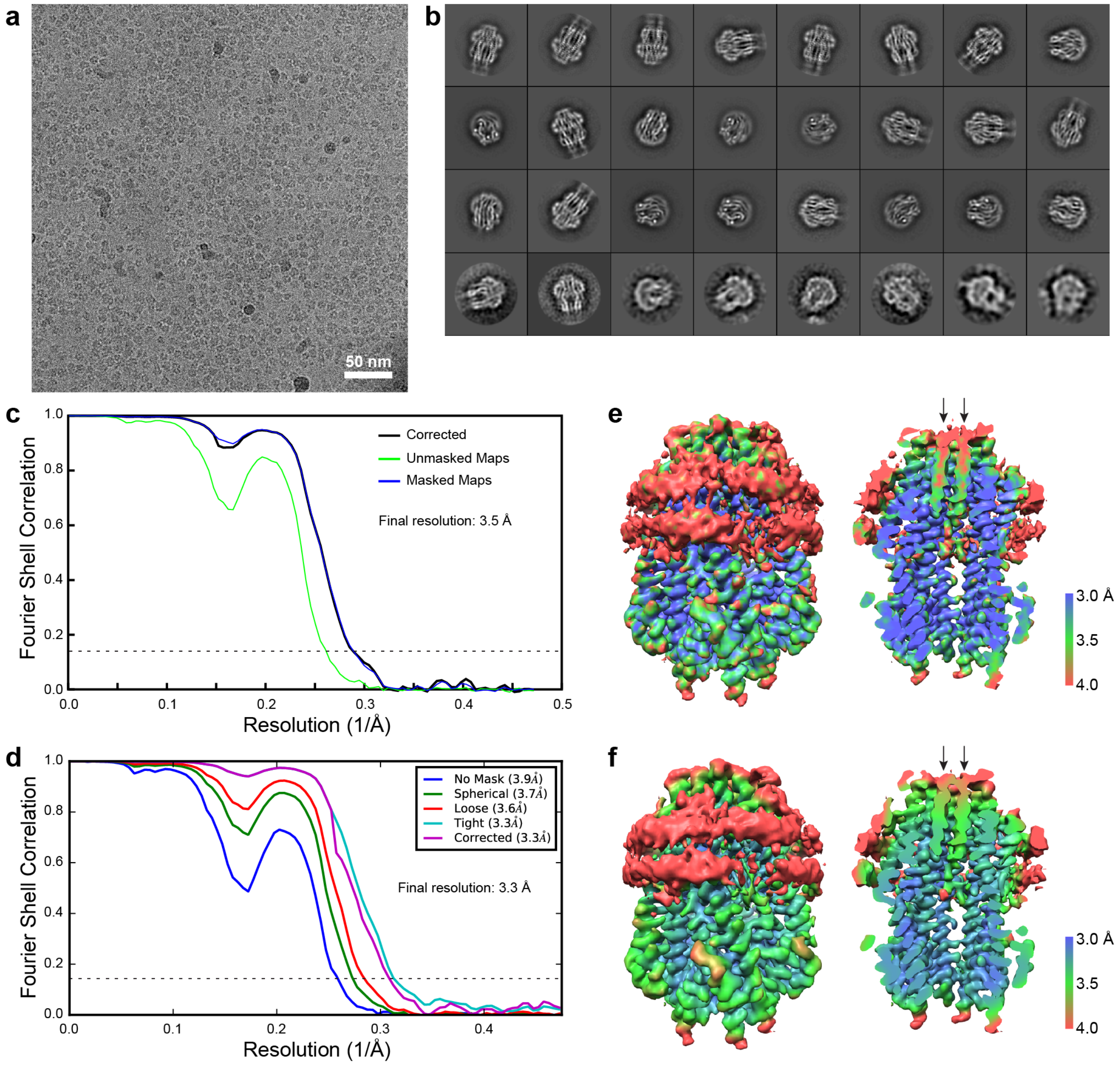

Resolution estimation of cryoEM single particle reconstructions of the ExbB-ExbD complex. a. Motion-corrected micrograph of the ExbB-ExbD sample at -2.2 µm defocus. b. Representative cryoEM 2D class averages of the ExbB-ExbD complex. Different angles of views ranging from the top view to side view exist in the data where the protein α-helices are dissectible. The side-length of the boxes is 20.4 nm. c and d. Fourier Shell Correlation curves from the final 3D autorefinement by RELION3 (c) and from the non-uniform refinement by cryoSPARC2 (d). e and f. Local resolution maps of the 3D reconstructions by RELION3 (e) and cryoSPARC2 (f). The local resolution maps are shown as surface view (left) and cross-sectional view (right). Two arrows point to two helices of ExbD in the pore of the ExbB pentamer. The local resolution maps are calculated by ResMap (e) and by cryoSPARC2 with an algorithm similar to the *blocres* program (f), respectively. The differences in resolution between (e) and (f) might result from the differences in the algorithms.

Supplementary Figure 4

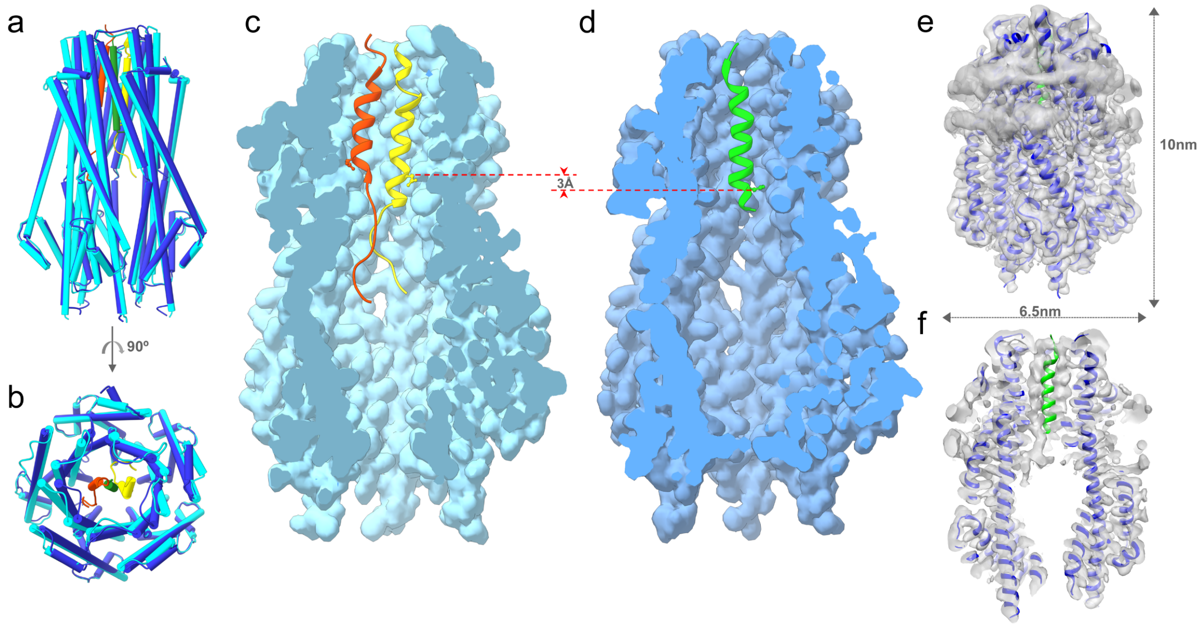

Comparison between the cryo-EM and crystal structures. a and b. Pipes and plank representation of the ExbB-ExbD cryo-EM structure (ExbB in cyan, ExbD in yellow and orange) and the crystal structure of ExbB-ExbD$_{deltaperi}$ (pdb 5SV1, ExbB in blue ExbD$_{deltaperi}$ in green). View perpendicular to the membrane (a) and from the periplasm (b). c and d. Slab through a surface representation of the pentamer of ExbB from cryo-EM (c) and crystallography (d). The structures of the N-terminal domains are shown in ribbon representation, with Asp25 represented as ball and stick. e and f. Overlay of the cryo-EM 3D map (grey surfaces) and the crystal structure (ExbB in blue, ExbD in green). f is a central section to show the differences between the ExbD TMs for the two structures. The dimensions of the complex are indicated.

Supplementary Figure 5

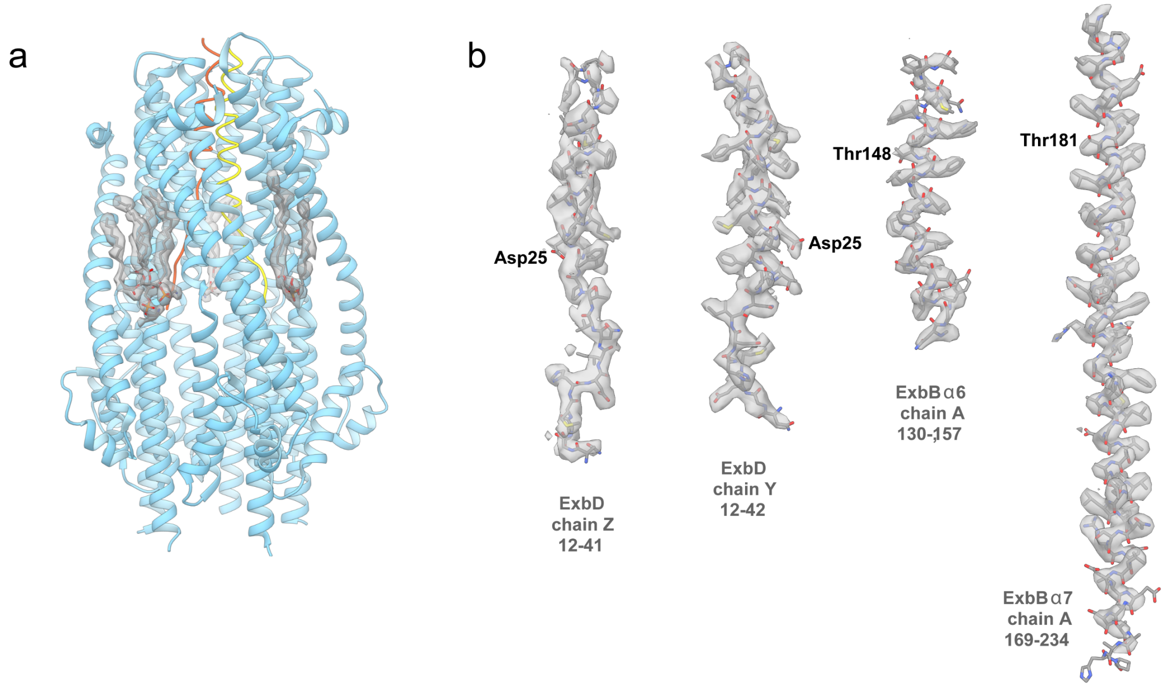

Representatives of cryo-EM density map. a. density maps and fitted atomic model structures of four phospholipids, superimposed on the cryo-EM structure of ExbB-ExbD shown with ribbon. b. density maps and fitted atomic models of the ExbD N-terminal and TM domains, and α6 and α7 helices of ExbB. The conserved Asp25 of ExbD and Thr148 and Thr181 of ExbB are indicated.
