# Supplementary material for: Cryo-EM structure of the bacterial Ton motor subcomplex ExbB–ExbD provides information on structure and stoichiometry
Source: Commun Biol. 2019 Oct 4;2:358. doi: 10.1038/s42003-019-0604-2 (PMC6778125; doi:10.1038/s42003-019-0604-2)
Supplement: Supplementary file 2 — Description of additional supplementary items [file 42003_2019_604_MOESM2_ESM.docx]

Description of additional supplementary items for:

**Cryo-EM structure of the bacterial Ton motor subcomplex ExbB-ExbD provides information on structure and stoichiometry**

Supplementary Movie 1. Periplasmic (top) view of ExbB X-ray and EM conformational changes.

Supplementary Movie 2. Side (membrane) view of ExbB X-ray and EM conformational changes.

Supplementary Movie 1


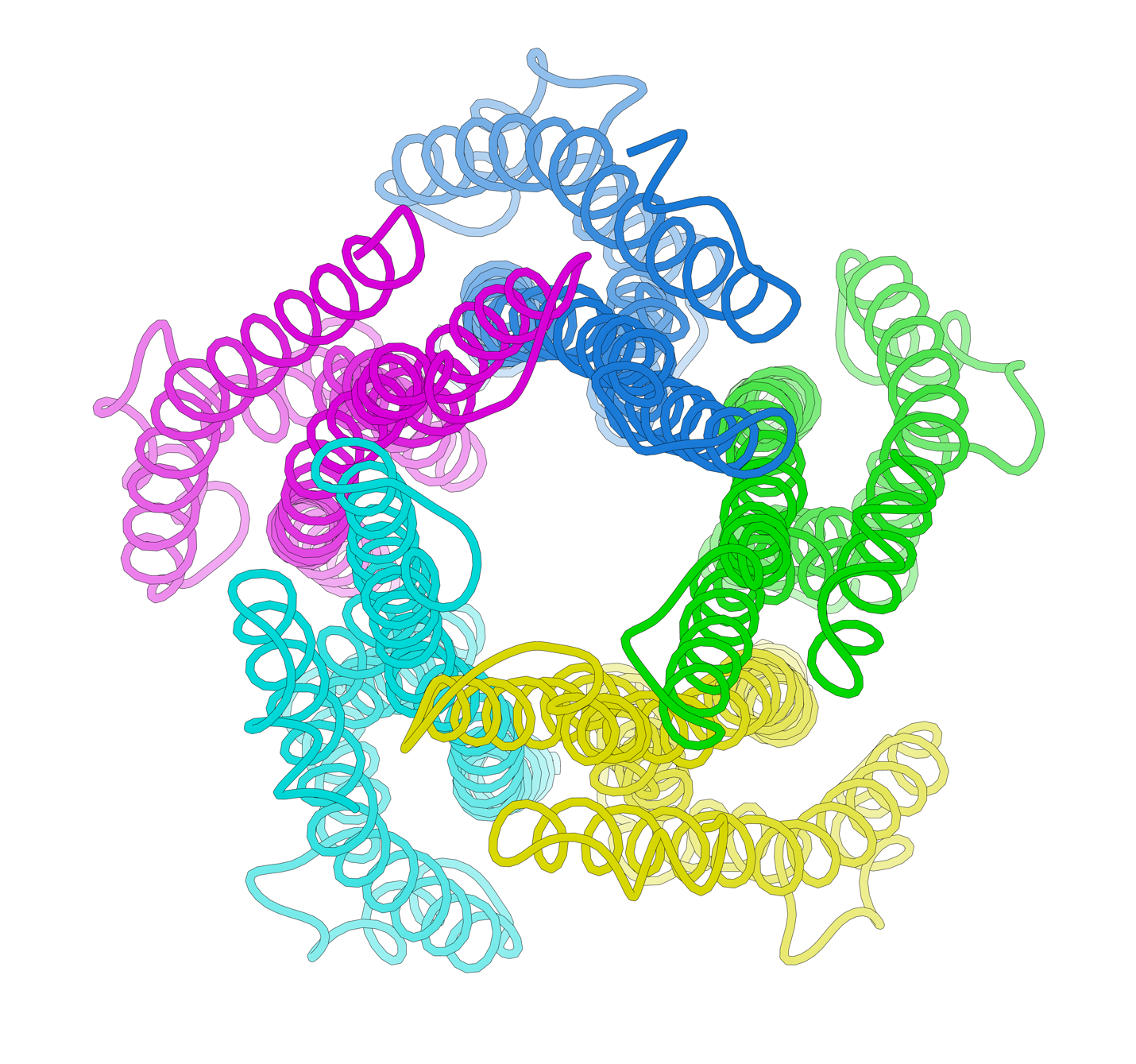


This morph movie shows changes in ExbB subunits, viewed from the periplasm, when comparing the crystal structure and the cryo-EM structure. Expansion of the ExbB pentamer accommodates two transmembrane helices of ExbD as seen in the cryo-EM structure.

Supplementary Movie 2


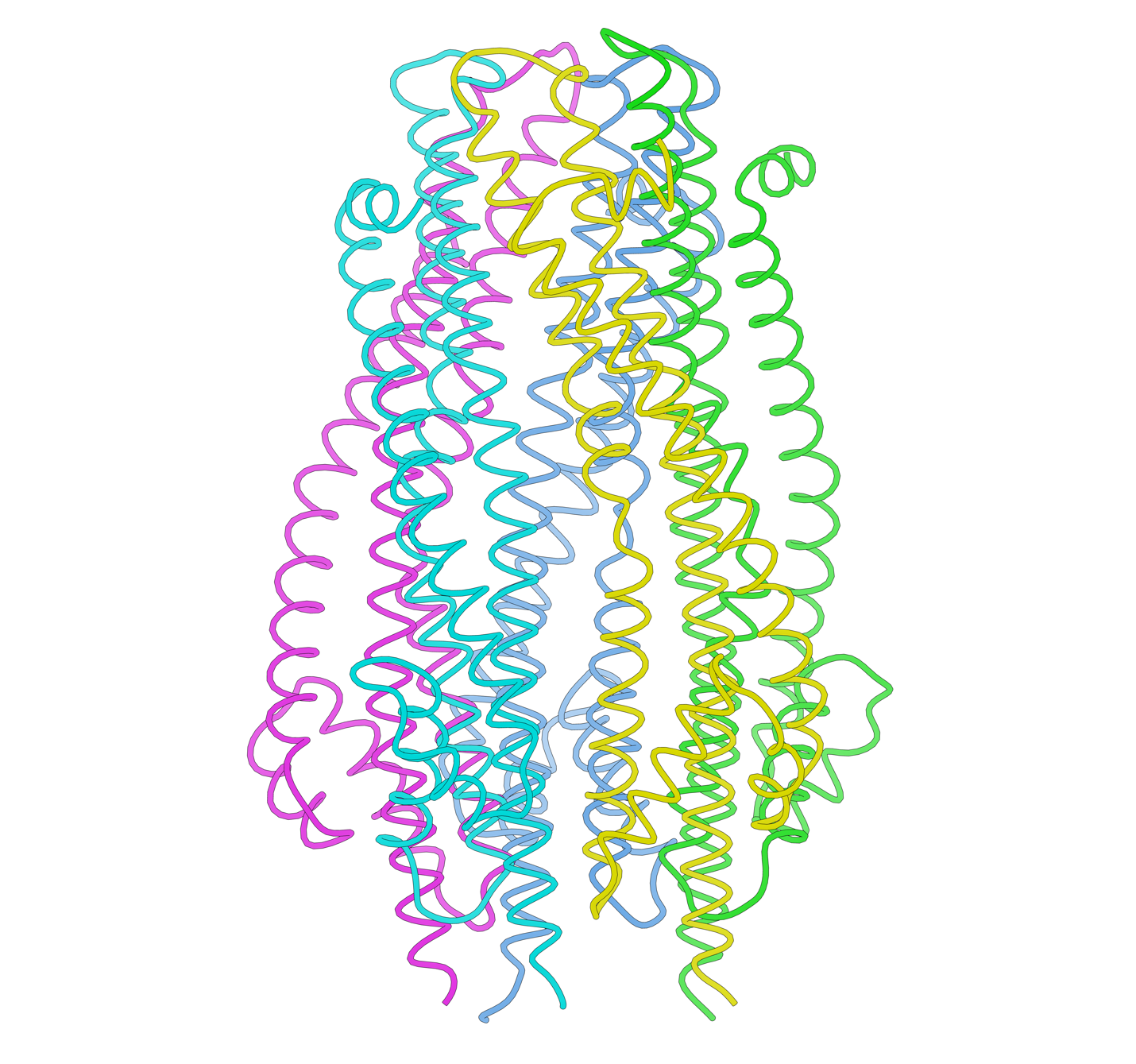


This morph movie shows changes in ExbB subunits, viewed from the membrane, when comparing the crystal structure and the cryo-EM structure. Expansion of the ExbB pentamer accommodates two transmembrane helices of ExbD as seen in the cryo-EM structure.
